# Supplementary material for: Microcin MccI47 selectively inhibits enteric bacteria and reduces carbapenem-resistant Klebsiella pneumoniae colonization in vivo when administered via an engineered live biotherapeutic
Source: Gut Microbes. 2022 Sep 29;14(1):2127633. doi: 10.1080/19490976.2022.2127633 (PMC9542533; doi:10.1080/19490976.2022.2127633)
Supplement: Supplemental Material [file KGMI_A_2127633_SM9939.docx]

**Microcin MccI47 selectively inhibits enteric bacteria and reduces carbapenem-resistant *Klebsiella pneumoniae* colonization *in vivo* when administered *via* an engineered live biotherapeutic**

**Authors: Benedikt M. Mortzfeld^1,2,*^, Jacob D. Palmer^3,4^, Shakti K. Bhattarai^1,2^, Haley L. Dupre^5^, Regino Mercado-Lubo^1^, Mark W. Silby^6^, Corinna Bang^7^, Beth A. McCormick^1,2^, Vanni Bucci^1,2,8,*^**

**Affiliations:**

^1^Department of Microbiology and Physiological Systems, University of Massachusetts Medical School, Worcester, MA, USA

^2^Program in Microbiome Dynamics, University of Massachusetts Medical School, Worcester, MA, USA

^3^Department of Zoology, University of Oxford, Oxford, United Kingdom

^4^Department of Biochemistry, University of Oxford, Oxford, United Kingdom

^5^Department of Bioengineering, University of Massachusetts Dartmouth, North Dartmouth, MA, USA

^6^Department of Biology, University of Massachusetts Dartmouth, Dartmouth MA, USA

^7^Institute of Clinical Molecular Biology, Christian-Albrechts-Universität zu Kiel, Kiel, Germany

^8^Program in Systems Biology, University of Massachusetts Medical School, Worcester, MA, USA

**Correspondence to:**

Vanni Bucci  [vanni.bucci2@umassmed.edu](mailto:vanni.bucci2@umassmed.edu)

Benedikt M. Mortzfeld [benedikt.mortzfeld@umassmed.edu](mailto:benedikt.mortzfeld@umassmed.edu)

**Supplementary Table 1**: Primers used for plasmid verification in Supplementary Figure 2B and quantitative PCR in Figure 2D.

| **Primer name** | **Sequence** | **Target** | **Fragment size** |
| --- | --- | --- | --- |
| pMut1_MobA_F | GTGCCCTGTTATCCAGGCTTATGG | pMut1 | 1208 bp |
| pMut1_ORF4_R | AGGTTGAAGGTCTCAGAGAATGAGAC |  |  |
| pMut2_ORF2_F | ATGTTAATCTGCTATTTGAATAGTCGAGTACGC | pMut2 | 1296 bp |
| pMut2_R | GCTCGTCATCGATCCGAATATTAATCG |  |  |
| pMut2_ORF2_F | ATGTTAATCTGCTATTTGAATAGTCGAGTACGC | pCure2-I47 | 1493 bp |
| pCure2_lacI_R | CGACATCGTATAACGTTACTGGTTTCAC |  |  |
| pMut2_ORF2_F | ATGTTAATCTGCTATTTGAATAGTCGAGTACGC | pMut2-I47 | 244 bp |
| pMut2_AmpR_R | TCTACACGACGGGGAGTCAGG |  |  |
| qPCR_gyrB_F | CATGGAGCGTCGTTATCCGA | gyrB | 147 bp |
| qPCR_gyrB_R | CTGCCGTGCTGTTCTTTGTC |  |  |
| qPCR_pMut2_F | CAAAGCCCCGAAATCATGCTC | pMut2 | 186 bp |
| qPCR_pMut2_R | CGGAGAAGTACGGCTTGTGG |  |  |


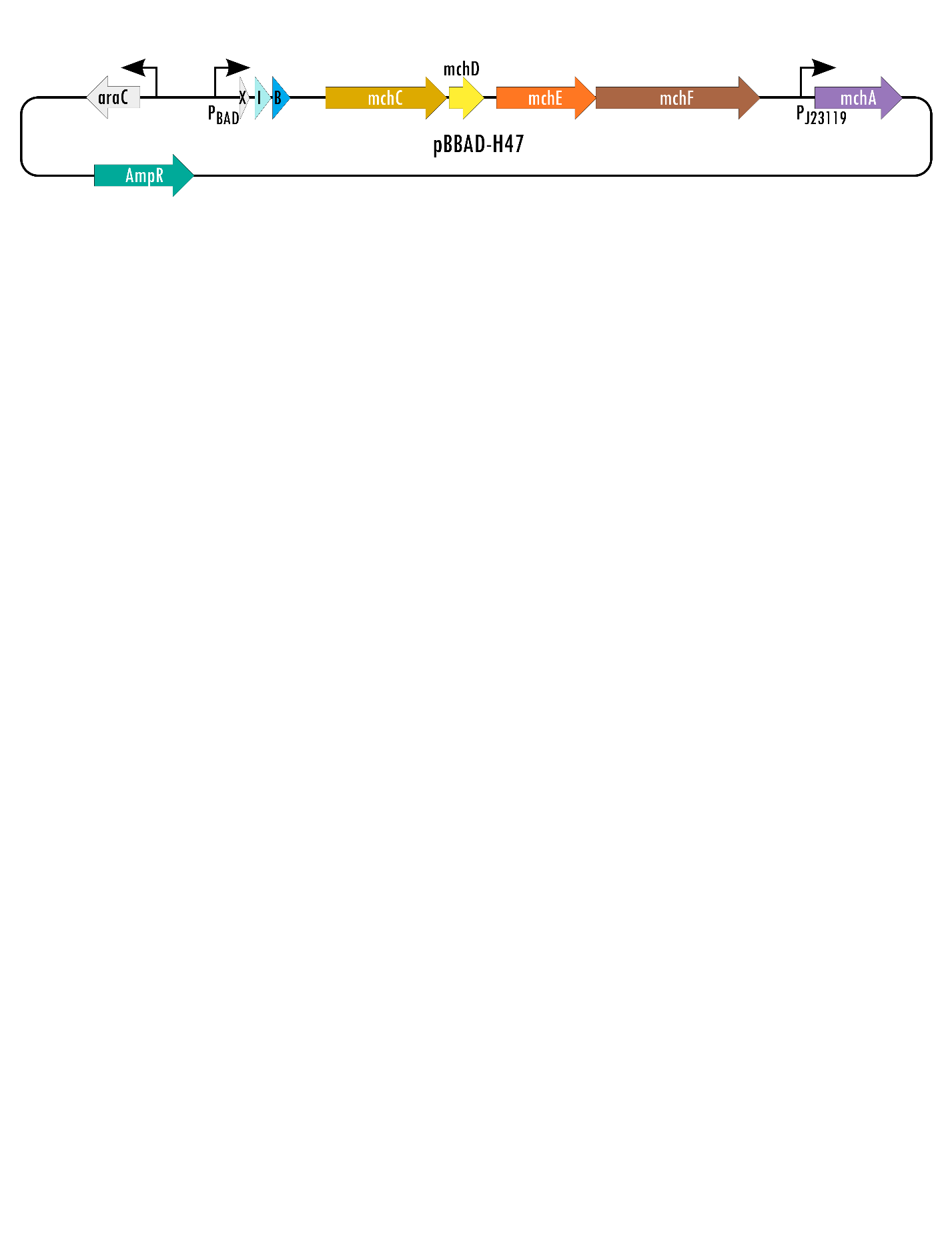


**Supplementary Figure 1**: Plasmid map of pBBAD-H47, a pUC19-based plasmid producing MccH47-MGE from mchB, the immunity peptide mchI under an L-arabinose inducible P_BAD_ promoter as well as the genes needed for post-translational modification mchCDEFA. AmpR = ampicillin resistance, X = mchX, I = mchI, B = mchB.


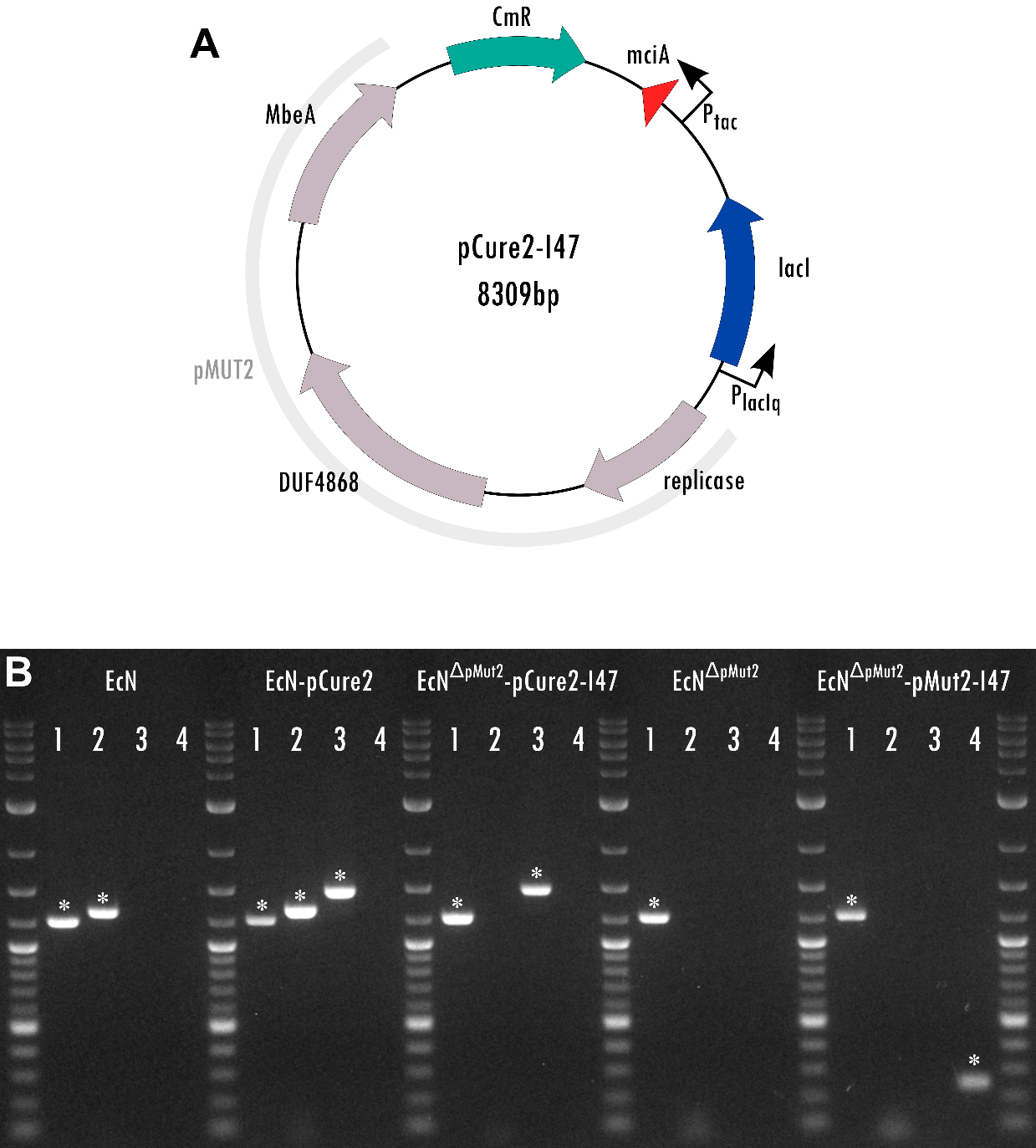


**Supplementary Figure 2**: (A) Plasmid map of pCure2-I47. Grey shaded area indicates the pMut2 backbone. CmR = chloramphenicol resistance. (B) Gel electrophoresis of PCR products for EcN strains created during the process pMut2 curing and transformation with pMut2-I47. (1) Primers against pMut1 (1208 bp), (2) primers against the insert-free pMut2 backbone (1296 bp), (3) primers against pCure2-I47 (1493 bp), (4) primers against pMut2-I47 (244 bp). Asterisks indicate specific fragments amplified by PCR. 1kb Plus DNA Ladder (New England Biolabs, Ipswich, MA).


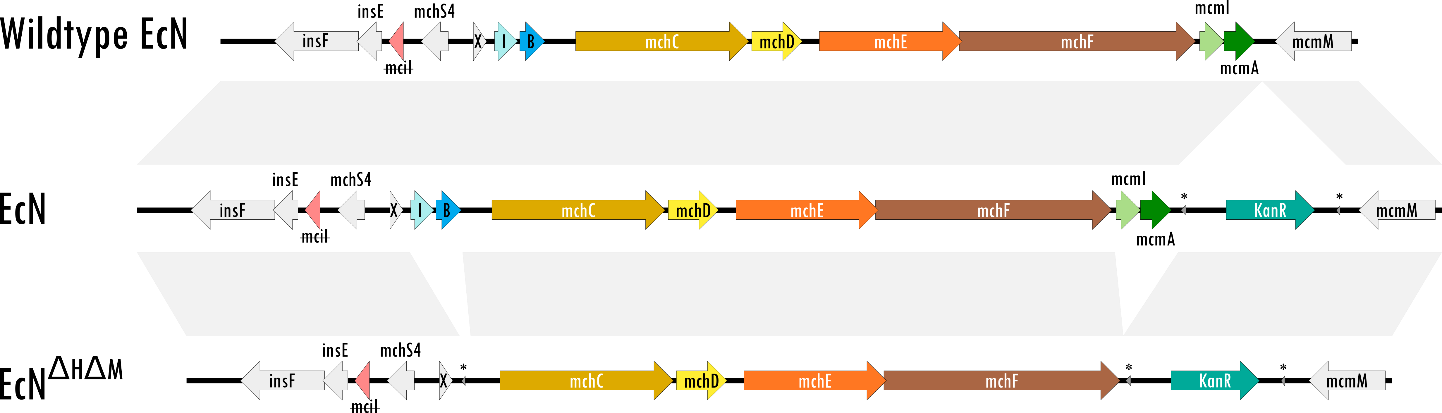


**Supplementary Figure 3**: Genetic organization of the E. coli Nissle 1917 class IIb microcin gene cluster. Wildtype EcN (top) harbors genes for production of the microcins MccH47 (mchB) and microcin MccM (mcmA) including their corresponding immunity genes mchI and mcmI. To allow selective colony counting, a kanamycin resistance cassette was introduced into the EcN genome and the resulting strain served as the EcN control in this study (center, EcN). A MccH47/MccM knockout strain (bottom, EcN^ΔH ΔM^) was generated to exclude the interference of native EcN microcin production, when assessing the potency of MccI47 against K. pneumoniae in vitro and in vivo. This strain served as the knockout control and the basis for the strain harboring the MccI47-producing plasmid pMut2-I47 (EcN^ΔH ΔM^-I47). Shaded areas indicate sequence similarity between the depicted gene clusters. X = mchX, I = mchI, B = mchB, ~~mciI~~ = truncated mciI, * = flippase recognition target (FRT) site, KanR = kanamycin resistance.


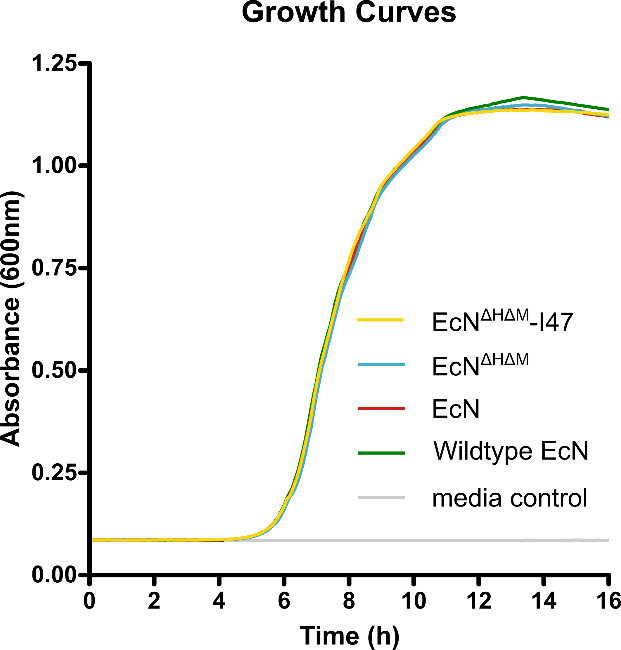


**Supplementary Figure 4:** Growth curves of different EcN strains. Note that none of the genetic modifications had an impact on the growth rate. Wildtype EcN represents the native strain, while all other strains were created for this study (see Supplementary Figure 3). EcN and EcN^ΔH ΔM^ serve as the study controls for EcN^ΔH ΔM^-I47, which is expressing MccI47 from pMut2-I47. n = 8.


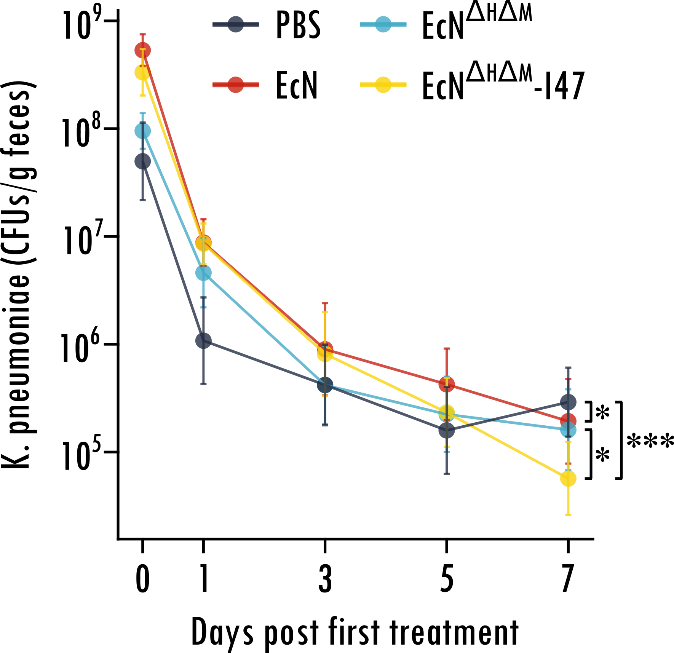


**Supplementary Figure 5:** Klebsiella pneumoniae (BAA 1705) colony forming units (CFUs) throughout the in vivo experiment. Linear mixed effect modeling. *: p < 0.05, ***: p < 0.001. n = 16.


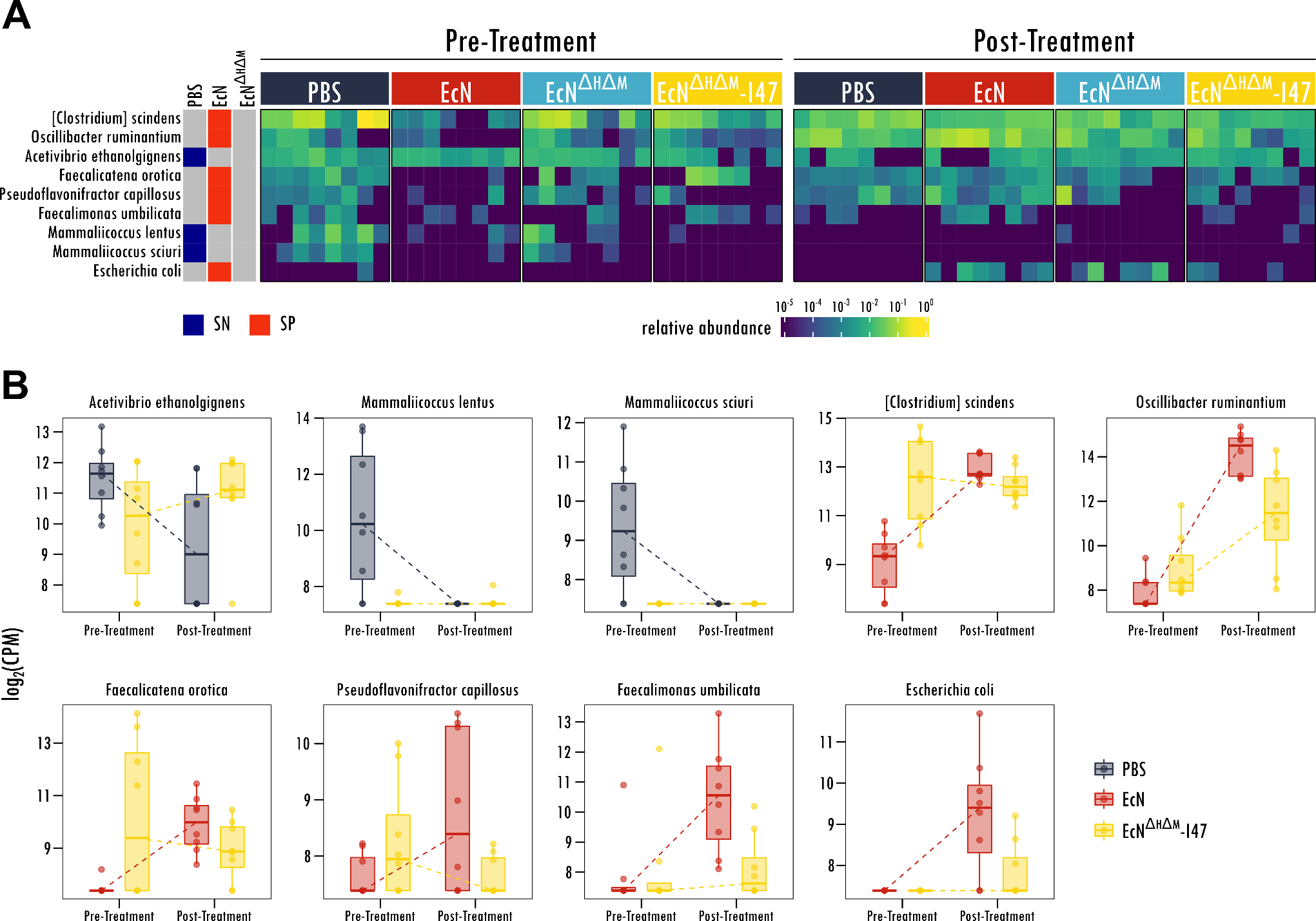


**Supplementary Figure 6:** Differential bacterial species analysis before and after treatment. (A) Differential species are significantly affected by the respective treatment compared to the baseline EcN^ΔH ΔM^-I47 (linear mixed effect modeling; Species_i_(counts)∼Time*Treatment+1|MouseID; p≤0.05). (B) Boxplots of affected species before and after treatment. SN = significant negative change, SP=significant positive change, CPM=counts per million.
